# Hydrogen gas mediates ascorbic acid accumulation and antioxidant system enhancement in soybean sprouts under UV-A irradiation

**DOI:** 10.1038/s41598-017-16021-0

**Published:** 2017-11-27

**Authors:** Li Jia, Jiyuan Tian, Shengjun Wei, Xiaoyan Zhang, Xuan Xu, Zhenguo Shen, Wenbiao Shen, Jin Cui

**Affiliations:** College of Life Sciences, Nanjing Agricultural University, Nanjing, Jiangsu 210095 China

## Abstract

The soybean sprout is a nutritious and delicious vegetable that is rich in ascorbic acid (AsA). Hydrogen gas (H_2_) may have potential applications in the vegetable processing industry. To investigate whether H_2_ is involved in the regulation of soybean sprouts AsA biosynthesis under UV irradiation, we set 4 different treatments: white light(W), W+HRW, UV-A and UV-A+HRW. The results showed that H_2_ significantly blocked the UV-A-induced accumulation of ROS, decreased TBARS content and enhanced SOD and APX activity in soybean sprouts. We also observed that the UV-A induced accumulation of AsA was enhanced more intensely when co-treated with HRW. Molecular analyses showed that UV-A+HRW significantly up-regulated AsA biosynthesis and recycling genes compared to UV-A in soybean sprouts. These data demonstrate that the H_2_ positively regulates soybean sprouts AsA accumulation under UV-A and that this effect is mediated via the up-regulation of AsA biosynthesis and recycling genes.

## Introduction

The soybean (*Glycine max* L.) sprout is the most popular vegetable in East Asia because of its high nutritional value and good taste^1^. This year–round vegetable is rich in bioactive ingredients, such as isoflavones, fatty acids, proteins and ascorbic acid^2^. Traditionally, soybean sprouts are cultivated in the absence of light. However, green soybean sprouts have appeared in the market and have become widely accepted due to their colour and good mouthfeel. In these green soybean sprouts, the synthesis of ascorbic acid, flavonoids and polysaccharides is significantly affected by light treatment^3^.

As one of the major light qualities, ultraviolet radiation (UV) has a prominent effect on the growth and development of plants^4,5^. The effect of UV radiation on plant growth has been reported by many investigators. For example, Albert *et al*.^6^ observed that UV radiation altered the plant morphology and architecture. Mazza *et al*.^7^ found that the response to UV includes increased damage to DNA, response of antioxidant and assembly of reactive oxygen species (ROS).

To cope with UV-induced oxidative stress, plant cells have evolved many highly capable defence systems with both non-enzyme and enzyme components. Antioxidant enzymes, like superoxide dismutase (SOD) and ascorbate peroxidase (APX), are mainly related with the subsistence of the cellular redox stable state in plant cells^8^. In addition to enzymatic antioxidant systems, non-enzymatic antioxidants, like ascorbic acid (AsA) and glutathione (GSH), appear in the aqueous phase (intracellular fluid)^9^.

**Figure 1:**
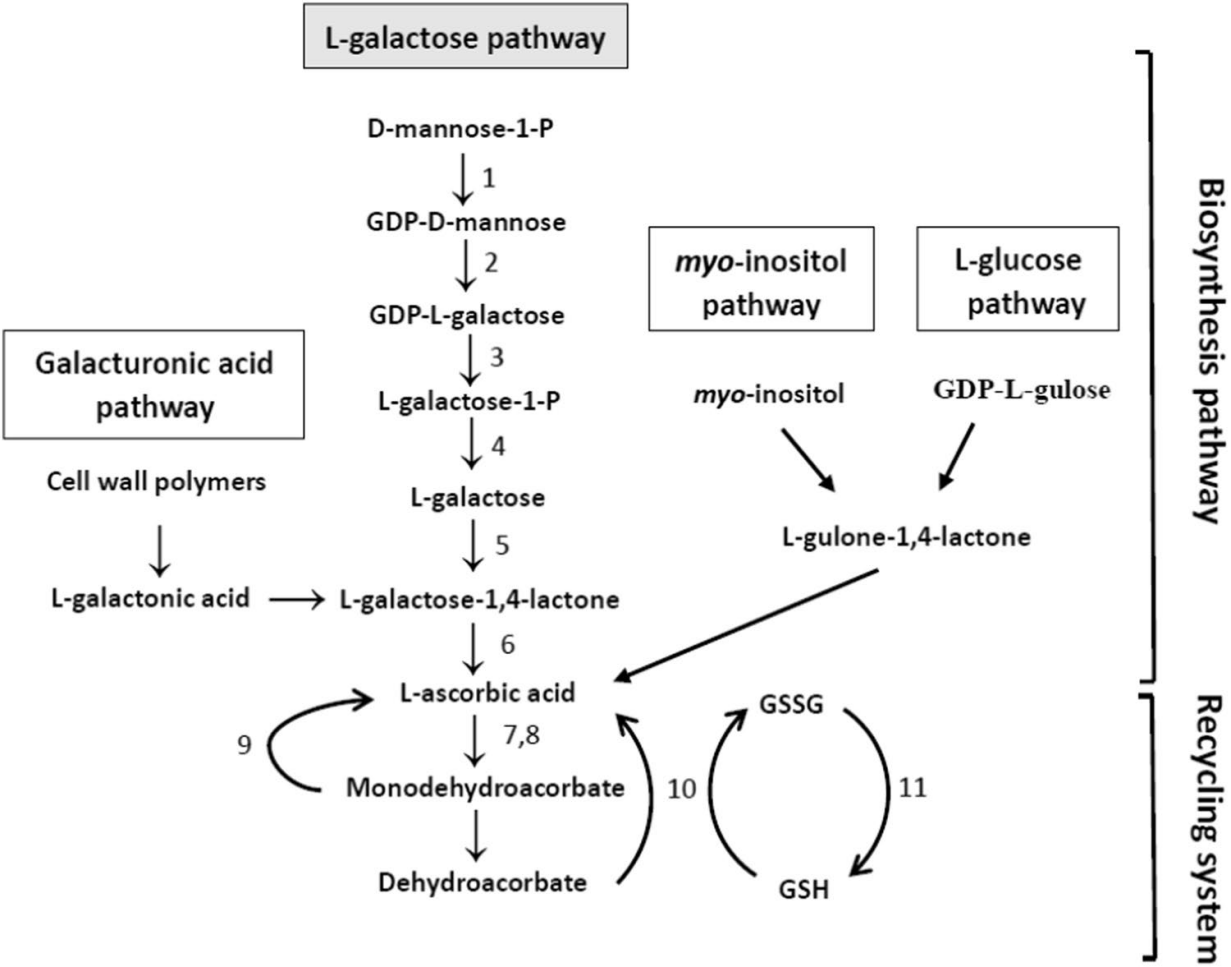
Possible schema for AsA accumulation in plants. Enzymes catalysing the numbered reactions are: (1) GDP-D-mannose pyrophosphorylase (GMP); (2) GDP-D-mannose-30,50-epimerase (GME); (3) GDP-L-galactose phosphorylase (VTC2); (4) L-galactose-1-phosphate phosphatase (VTC4);(5) L-galactose dehydrogenase (GDH); (6) L-galactono-1,4-lactone dehydrogenase (GLDH); (7) ascorbate oxidase (AO); (8) ascorbate peroxidase (APX);(9) monodehydroascorbate reductase (MDHAR); (10) dehydroascorbate reductase (DHAR); (11) glutathione reductase (GR).

As an important antioxidant, AsA responds to light, especially short wavelengths of light, such as UV-A^10–12^. An adequate level of AsA is essential for the different jobs of scavenging reactive oxygen species under UV-A^13^. As shown in Fig. 1, four pathways of AsA synthesis in plants have been proposed, including the L-galactose or Smirnoff-Wheeler (SW), D-galacturonic acid, L-glucose, and myo-inositol^14,15^. Among these proposed pathways, the L-galactose pathway is regarded as the major pathway for biosynthesis of AsA in plants^16^. All the concerned genes that encode enzymes that are required in this pathway have been characterized, including GDP-D-mannose-30,50-epimerase (GME), GDP-D-mannose pyrophosphorylase (GMP), GDP-L-galactose phosphorylase (VTC2), L-galactose-1-phosphate phosphatase (VTC4), L-galactose dehydrogenase (GDH) and L-galactono-1,4-lactone dehydrogenase (GLDH)^17^. When AsA is formed, it is not stable *in vivo* and can be oxidized to dehydroascorbate (DHA) and monodehydroascorbate (MDHA) via ascorbate peroxidase (APX) and ascorbate oxidase (AO). These oxidized forms can then be reduced to AsA by dehydroascorbate reductase (DHAR) and monodehydroascorbate reductase (MDHAR), respectively. The accumulation of AsA in plant tissues is regulated by an effective balance between biosynthesis and recycling^18^.

Hydrogen gas (H_2_), a reducing gas, has been listed as a safe food additive in many countries. Recent research has shown that H_2_ can function as a unique useful gaseous molecule in plant adaptive responses to abiotic stress^19,20^. Su *et al*.^21^ reported that UV-A induced toxicity was reduced by exogenously applied hydrogen-rich water (HRW) through the regulation of anthocyanin synthesis and reestablishment of ROS homeostasis in radish sprouts. Jin *et al*.^22^ found that H_2_ might act as an essential gaseous molecule that relieves oxidative stress through HO-1 signalling.

Soybean sprouts are one of the most common and economic vegetables in East Asian countries owing to its high nutritional value and simple production method. Increasing the quality and nutritional value of soybean sprouts is becoming an issue^23^. Our objective is to find efficacious and safe methods to increase nutritional value of soybean sprouts. As a reducing gas, whether H_2_ has a particular function in the modulation of UV-A-induced accumulation of AsA is clearly unknown. Thus, the objective of the current study was to study the effect of H_2_ on the AsA content of soybean sprouts under UV-A irradiation. Secondly, we assessed the possible mechanism of H_2_ affecting the ROS and TBARS levels, key enzymes involved in oxidative metabolism, and AsA biosynthesis-related genes.

## Results

### HRW alleviated inhibition of hypocotyl elongation induced by UV-A and enhanced accumulation of AsA and AsA+DHA in the soybean sprouts

**Figure 2:**
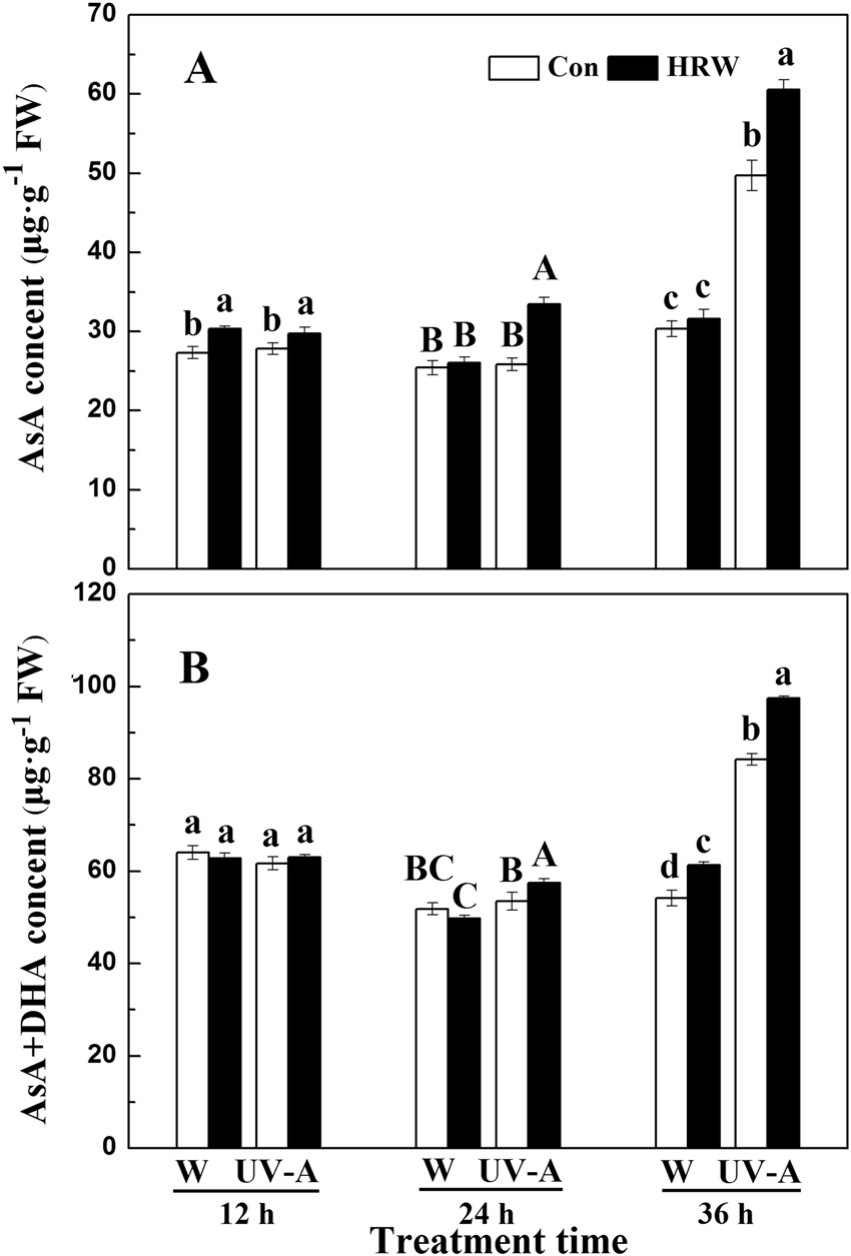
Changes in AsA (**A**) and AsA+DHA (**B**) accumulation according to different treatments of soybean sprouts. Plants were grown in the dark for 72 h and then transferred to 50 ± 5 µmol·m^−2^·s^−1^ white light or 5.5 W·m^−2^ UV-A light for the times indicated prior to harvest. Con: Sprouts cultivated in distilled water; HRW: Sprouts cultivated in HRW; W: Sprouts cultivated under white light; UV-A: Sprouts cultivated under UV-A. Data are the mean ± standard error (n = 3); Different letters indicate significant differences (Duncan’s test, *P* < 0.05).

The development of soybean sprouts with different treatments for 36 h is shown in Supplemental Fig. 1, and the root length, hypocotyl length, total weight, fresh edible weight and fresh edible rate were determined. As shown in Supplemental Table 1, UV-A irradiation significantly induced the inhibition of hypocotyl elongation, the total fresh weight and edible weight can be alleviated by HRW. However, the root length and edible rate were not influenced. As expected, at 36 h, the AsA content of soybean sprouts under UV-A was very high, and HRW significantly enhanced this effect (Fig. 2A). When treated with UV-A+HRW, the AsA content of soybean sprouts was approximately 60–70 µg·g^−1^ FW at 36 h. The variation tendency of AsA+DHA was the same as AsA (Fig. 2B). These results showed that UV-A significantly increased the AsA and AsA+DHA content in soybean sprouts and that HRW further increased the AsA and AsA+DHA content.

### The H_2_ concentration of HRW was affected by soybean sprouts and treatment conditions

**Figure 3:**
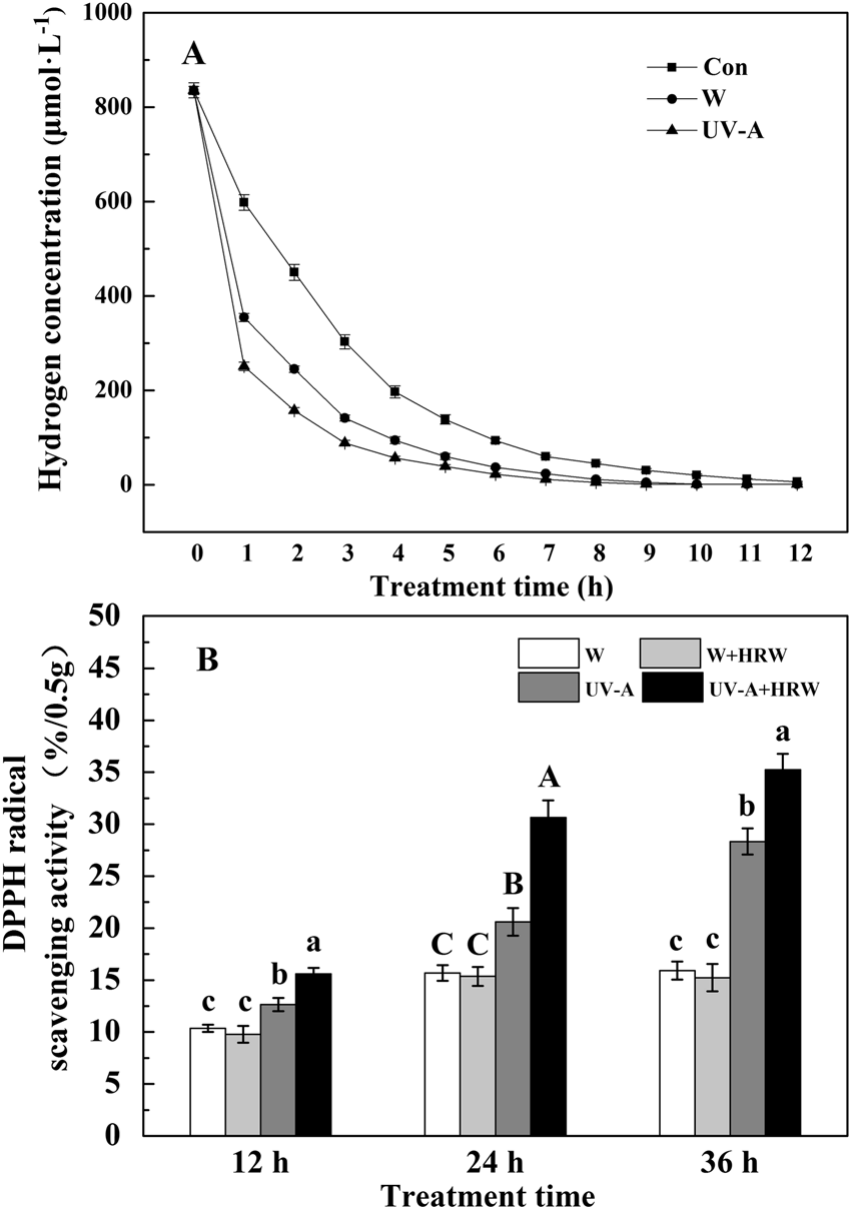
Effects of the different treatment on H_2_ concentrations of HRW (**A**) and the DPPH scavenging effect (%) in soybean sprouts (**B**). A Con: HRW without soybean sprouts; W:HRW with soybean sprouts under white light; UV-A: HRW with soybean sprouts under UV-A light. (**B**) Sprouts were grown in the dark for 72 h and then transferred to 50 ± 5 µmol·m^−2^·s^−1^ white light or 5.5 W·m^−2^ UV-A light for the times indicated prior to harvest. W: Sprouts cultivated in distilled water under white light; W+HRW: Sprouts cultivated in HRW under white light; UV-A: Sprouts cultivated in distilled water under UV-A; UV-A+HRW: Sprouts cultivated in HRW under UV-A. Error bars represent SD among three replicates, and different letters indicate significant differences (Duncan’s test, *P* < 0.05).

To provide support for the hypothesis that H_2_ in the water can be absorbed by the plant, we designed the following test: saturated HRW without planting soybean sprouts was used as the control, and the experimental group included saturated HRW planted with soybean sprouts irradiated with white light or UV-A irradiation. The H_2_ concentration of HRW was measured every 1 h after light treatment. As shown in Fig. 3A, the H_2_ concentration of HRW decreased faster than that of the control under both white light and UV-A irradiation. The H_2_ concentration of saturated HRW was 829 μmol·L^−1^ at 0 h, and it decreased slowly in the control. After 1 h, the H_2_ concentration of HRW was 550–650 μmol·L^−1^ in the control and was 200–400 μmol·L^−1^ in the experimental group. Among these conditions, the H_2_ concentration decreased rapidly under UV-A irradiation and was 200–300 μmol·L^−1^. For 2–12 h, the H_2_ concentration decreased slowly in the experimental group but was faster compared to the control. Above all, H_2_ in HRW was absorbed by soybean sprouts directly, which decreased the H_2_ concentration in HRW. Additionally, the absorbance rate was the highest under UV-A irradiation.

### DPPH radical scavenging activity of soybean sprouts extract was increased by UV-A and HRW enhanced this effect

The DPPH radical scavenging activity was measured. As shown in Fig. 3B, with an increase in treatment time, the DPPH radical scavenging activity was significantly increased. Exposure to UV-A for 12, 24 or 36 h significantly increased the DPPH radical scavenging activity, and HRW treatment further improved the scavenging activity, especially at 36 h, when the DPPH radical scavenging activity was highest for the UV-A+HRW treatment. The amount of scavenging activity was nearly 2-fold higher as compared to that observed under white light. However, HRW had no significant effect on the DPPH radical scavenging activity under white light.

### HRW alleviated UV-A-induced oxidative damage and increased antioxidant enzyme activity in soybean sprouts

**Figure 4:**
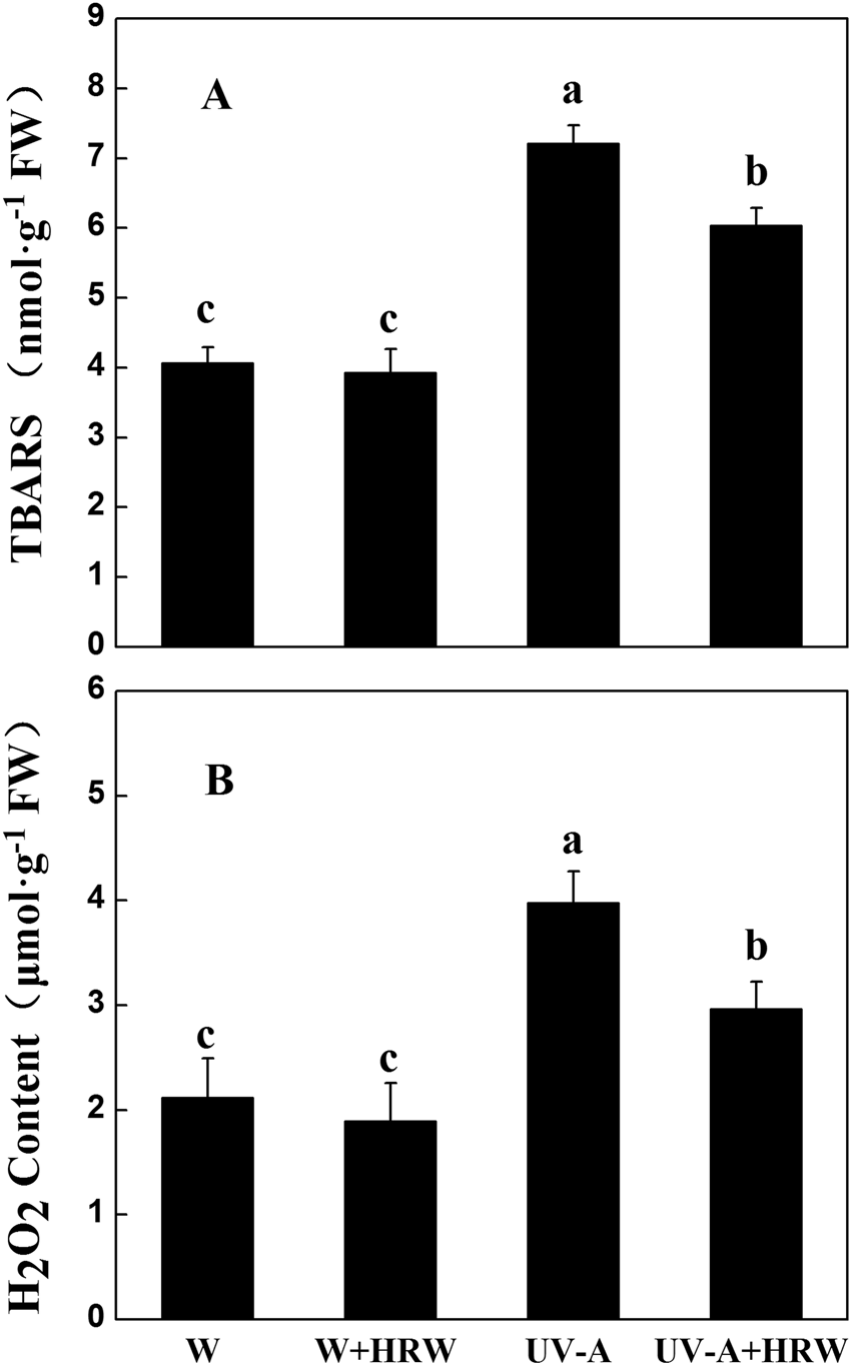
Effects of different treatments on TBARS (**A**) and H_2_O_2_ (**B**) content in soybean sprouts. Sprouts cultivated in distilled water (H_2_O) or HRW were exposed to white light or UV-A for 36 h. W: Sprouts cultivated in distilled water under white light; W+HRW: Sprouts cultivated in HRW under white light; UV-A: Sprouts cultivated in distilled water under UV-A; UV-A+HRW: Sprouts cultivated in HRW under UV-A. Error bars represent SD among three replicates. Measurements in the same column followed by different letters are significantly different at *P* < 0.05.

Abiotic stress is thought to cause oxidative damage to plants. To study the HRW influence on soybean sprout oxidative damage, we determined the content of TBARS and H_2_O_2_ as shown in Fig. 4. Compared to W treatment, UV-A significantly increased the TBARS content of soybean sprouts. HRW significantly reduced the TBARS content of soybean sprouts under the UV-A treatment in comparison to the W treatment. These phenomena showed that HRW can relieve the oxidative damage caused by UV-A.

Similar to TBARS, UV-A treatment significantly increased the content of H_2_O_2_ compared to W, but HRW significantly reduced the H_2_O_2_ content caused by UV-A (Fig. 4B). However, the H_2_O_2_ content was still higher than W under UV-A+HRW treatment.

**Figure 5:**
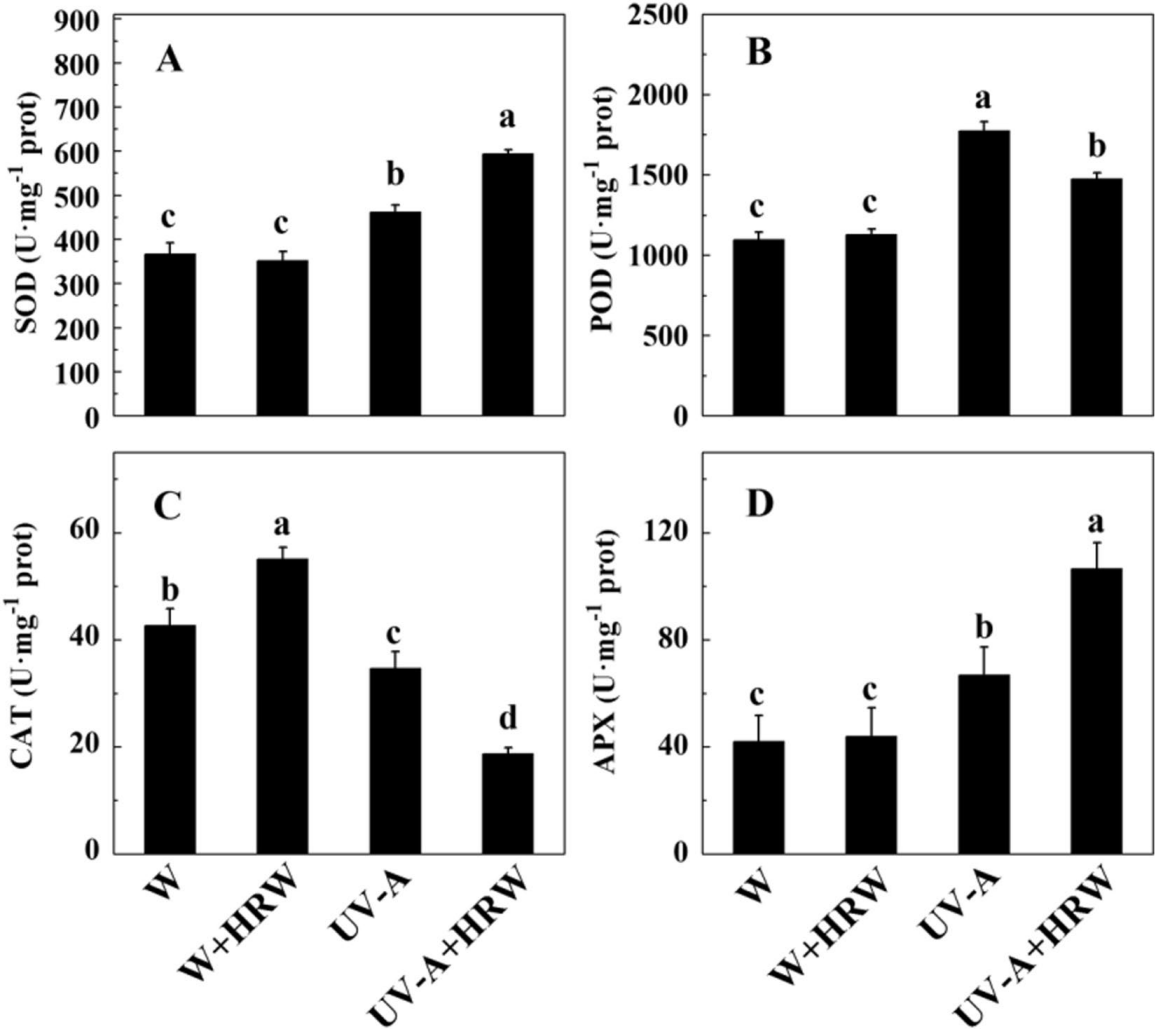
Effects of different treatment on SOD (**A**), POD (**B**), CAT(**C**) and APX (**D**) activities in soybean sprouts. Sprouts cultivated in distilled water (H_2_O) or HRW were exposed to white light or UV-A for 36 h. W: Sprouts cultivated in distilled water under white light; W+HRW: Sprouts cultivated in HRW under white light; UV-A: Sprouts cultivated in distilled water under UV-A; UV-A+HRW: Sprouts cultivated in HRW under UV-A. Error bars represent SD from three replicates. Measurements in the same column followed by different letters are significantly different at *P* < 0.05.

To further reveal the mechanism through which HRW relieved soybean sprout oxidative damage, we measured the enzyme activity of SOD, POD, CAT and APX. As shown in Fig. 5, UV-A significantly enhanced the enzyme activity of SOD and APX, and HRW further increased the enzyme activity of SOD and APX (Fig. 5A,D). Compared to W treatment, SOD and APX activity increased by 0.5 and 1.5 times, respectively, under UV-A+HRW treatment. Compared to W, POD activity also increased significantly (Fig. 5B), but CAT activity dropped markedly under UV-A treatment. The activity of CAT reached a minimum under UV-A+HRW treatment (Fig. 5C), which was approximately one-third of the activity from the W+HRW treatment.

### HRW up-regulated the expression level of AsA biosynthesis-related genes in the soybean sprouts

**Figure 6:**
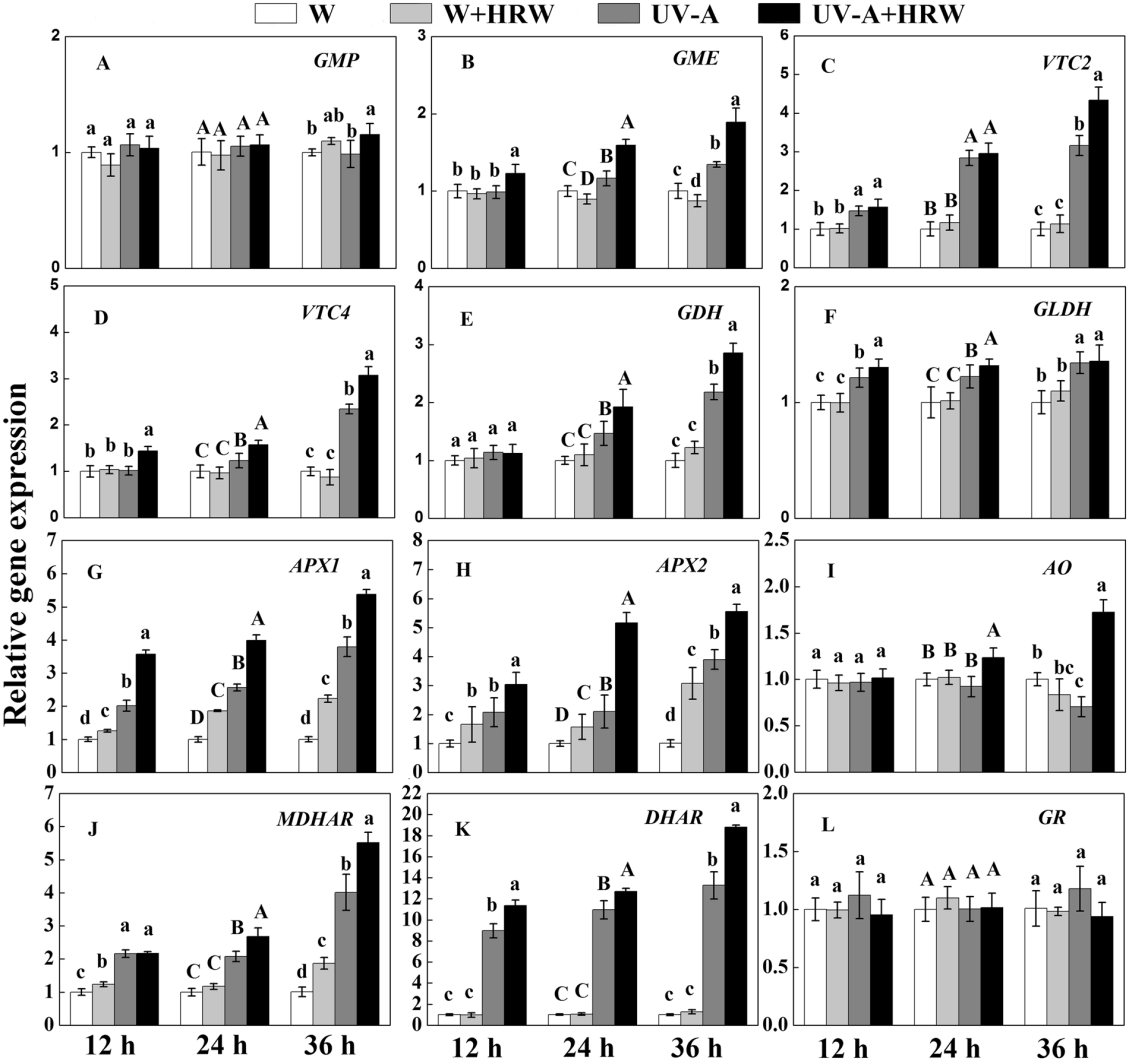
Transcript abundance of the GMP (**A**), GME (**B**), VTC2 (**C**), VTC4 (**D**), GDH (**E**), GLDH (**F**), APX1 (**G**), APX2 (**H**), AO (**I**), MDHAR (**J**), DHAR (**K**) and GR (**L**) expression in the soybean sprouts exposed to white light or UV-A for 12 h, 24 h and 36 h. Plants were grown in the dark for 72 h and then transferred to 50 ± 5 µmol·m^−2^·s^−1^ white light or 5.5 W·m^−2^ UV-A light for the times indicated prior to harvest. W: Sprouts cultivated in distilled water under white light; W+HRW: Sprouts cultivated in HRW under white light; UV-A: Sprouts cultivated in distilled water under UV-A; UV-A+HRW: Sprouts cultivated in HRW under UV-A. Data are the mean ± standard error (n = 3); Columns represent the mean (+SD) values of independent experiments (n = 3) based on transcript-level data normalized to W+Con at 12 h, 24 h and 36 h; Different letters indicate significant differences (Duncan’s test, *P* < 0.05).

To further investigate the mechanism of the increase of AsA induced by HRW, quantitative real-time PCR analyses were conducted on soybean sprouts exposed to white light or UV-A for 12, 24 and 36 h. As shown in Fig. 6, in the biosynthesis pathway at 12 h and 24 h, the transcript levels of *GMP* were not significantly affected, but at 36 h, the transcript levels of *GMP* were significantly increased by UV-A+HRW treatment (Fig. 6A). The transcript levels of *GME* were significantly affected by different treatments, and exposure to UV-A for 24 or 36 h significantly increased the transcription levels of *GME* compared to white light exposure, HRW also up-regulated the transcription levels of *GME* (Fig. 6B). The transcription levels of *VTC2*, *VTC4* and *GDH* showed a similar tendency to that of *GME*. Among them, the transcription levels of *VTC2*, *VTC4* and *GDH* were intensely up-regulated at 36 h by UV-A+HRW treatment (Fig. 6C,D,E). The transcription levels of *GLDH* did not change much under different treatments. However, at varying treatment times, the transcription levels of *GLDH* with UV-A treatment were higher compared to white light (Fig. 6F).

In the recycling pathway, exposure to UV-A for 12, 24 or 36 h significantly increased the transcription levels of *APX1* compared to white light. HRW enhanced this effect, especially at 36 h, when the transcript levels of *APX1* were highest (Fig. 6G). The transcript level tendencies of *APX2*, *MDHAR* and *DHAR* were similar to *APX1* (Fig. 6H,J,K). At 12 and 36 h, the transcription levels of *AO* were not significantly affected by different treatments, but with UV-A+HRW treatment, the transcription levels of *AO* increased at 36 h (Fig. 6I). The different treatments had no significant effect on the GR transcript levels (Fig. 6L).

## Disscussion

In traditional planting of green soybean sprouts, white light is used as the light source^3,24^. Recently, some studies found that short wavelength light like UV-A was beneficial to increase health-promoting compounds in sprouts, such as flavonoids, rutin^24–26^. Our previous study showed that UV-A can significantly increase anthocyanin content in soybean sprouts^1,27^. But UV-A can cause oxidation damage, decrease the hypocotyl length and yields in soybean sprouts, which is not conducive to the industrial production of soybean sprouts^27,28^. As reducing gas, the role of H_2_ in plant physiology, especially in abiotic stress, has attracted the attention of many researchers. Xie *et al*. reported on the H_2_ participation in enhancing tolerance to salt in Arabidopsis seedlings^29^. Jin *et al*. found that H_2_ was able to act as a unique bioactive molecule in improving plant tolerance to oxidative stress^22^. Whether can H_2_ relieve oxidation damage and yield decline caused by UV-A in soybean sprouts has not been reported, therefore, we set four treatment: W, W+HRW, UV-A, UV-A+HRW. Our result shows that H_2_ can significantly release the inhibition of hypocotyl elongation induced by UV-A and boost fresh weight in soybean sprouts, meanwhile, HRW can obviously ease the oxidation damage caused by UV-A and increase AsA content in soybean sprouts.

H_2_ has a great antioxidant capacity^30^. Recently, some studies found that H_2_ could enhance the tolerance of plants to multiple environmental stresses, including drought and salt stress^31–33^. Same to H_2_, AsA also has a great antioxidant capacity because it is a natural oxidant scavenger and a primary reducing agent in biochemical processes^34^. Did the exogenous hydrogen donor have some effect on the AsA content of soybean sprouts under UV-A? This question prompted us to investigate the effects of HRW on the AsA contents in soybean sprouts. As expected, our results showed that the AsA and AsA+DHA contents in the soybean sprouts under UV-A treatment increased significantly in comparison to the W treatment (approximately 1.5-fold) (Fig. 2). What’s more, the AsA and AsA+DHA accumulations under UV-A+HRW was higher than UV-A single treatment. This indicates that AsA content can be significantly increased by adding exogenous H_2_. Our results suggest that H_2_ may via increased AsA content enhance the tolerance of soybean sprouts to UV-A irradiation. For better understanding of the relationship between AsA biosynthesis and HRW, AsA biosynthesis-related genes were identified. We found that HRW not only increased the expression level of AsA biosynthesis genes, such as *GME*, *VTC2*, *VTC4* and *GDH* (Fig. 6B–E), but also up-regulated the transcript level of recycling genes, including *APX1*, *APX2*, *AO*, *MDHAR* and *DHAR* (Fig. 6G–K). We speculate that H_2_ may influence MYB or DOF transcriptional factors to increase the expression of AsA biosynthesis-related genes^35,36^, this hypothesis needs further complementary genetic approaches to support our findings.

As we all know, UV-A irradiation can induce the production of reactive oxygen species (ROS), which can damage plant cells. To protect the plants from oxidative stress, plants have enzymatic scavengers such as SOD, POD, CAT, APX^21,37–39^. Our result show that HRW can significantly increase the activity of SOD and APX (Fig. 5A,D). Higher SOD and APX enzyme activity is beneficial for plants to resist UV-A, meanwhile, HRW can boost the DPPH free radical scavenging capacity of soybean sprouts (Fig. 3B). This effect caused us to hypothesize that H_2_ improves the tolerance of soybean sprouts to UV-A. To authenticate this hypothesis, the H_2_O_2_ and TBARS contents in the soybean sprouts were measured. As expected, HRW decreased the H_2_O_2_ and TBARS content in soybean sprouts under the combination of UV-A+HRW compared to sprouts treated with UV-A alone (Fig. 4). These results indicate that H_2_ can alleviate the oxidant damage caused by UV-A and enhance antioxidative ability. Similar results were also observed by Wu *et al*. and Jin *et al*.^22,40^, demonstrate that H_2_ treatment can decrease TBARS and ROS production in Chinese cabbage under cadmium stress^40^, and alfalfa leaves under paraquat stress^22^. Thus, our results indicate that exogenous H_2_ treatment, which is easy to use, safe, and economical, on soybean sprouts production may be a good option to alleviate UV-A induced yield reduction and increase AsA content in soybean sprouts.

In conclusion, we report that H_2_ alleviated the inhibition of hypocotyl elongation caused by UV-A, and increased the fresh weight of soybean sprouts (Supplemental Table 1). While, H_2_ decreased UV-A-induced oxidative damage and improved the AsA content of soybean sprouts under UV-A irradiation (Fig. 2). A possible mechanism may be that H_2_ increases the activity of SOD and APX and reduces the ROS and TBARS content of soybean sprouts (Figs 4 and 5). At the same time, qRT-PCR analysis indicates that H_2_ significantly up-regulates AsA biosynthesis and recycling genes (Fig. 6). These results suggests that HRW could probably be applied in the soybean sprout industry or commercially, after further research and assessments on quality and safety of food.

## Methods

### Plant material and growth conditions

Soybean (*Glycine max* L.) seeds were immersed in distilled water for 12 h and then transferred to petri dishes germinated for 36 h at 25 °C ± 1 °C. Uniform germinated seeds were selected and cultured in plastic chambers with distilled water or HRW. Four treatments with 3 replications each were set: white light(W), W+HRW, UV-A, and UV-A+HRW. Soybean sprouts were cultured in the darkness for 36 h in the greenhouse at 25 °C ± 1 °C. Then soybean sprouts were grown in growth chambers (Safe Instrument Experimental Factory Zhejiang, China) with white light or UV-A lamps (Philips, Amsterdam, Netherlands) which emit ultraviolet rays between 350 nm and 400 nm with a peak of 360 nm. The intensity of white light was set at 50 ± 5 µmol·m^−2^·s^−1^, and the UV-A dosage was set at 5.5 W·m^−2^.

### Preparation of HRW

Hydrogen gas (99.99%, v/v) produced from a hydrogen gas generator (SHC-300; Saikesaisi Hydrogen Energy Co., Ltd., Shandong, China) was bubbled into 1000 ml distilled water (pH 6, 25 °C) at the rate of 550 mL·min^−1^ for 20 min to reach saturation condition.

### Measuring H_2_ concentration of HRW

The H_2_ concentration of HRW was calculated using a needle-type Hydrogen Sensor (Unisense, Denmark) according to the manufacturer’s instructions. The H_2_-specific electrode had a tip with a diameter of 50 µm and was polarized for 4 h prior to use. While performing electrode analysis, this needle was placed in HRW. After the basal line of the H_2_ signal was stable, the corresponding data was recorded. A standard solution of HRW was made by saturating H_2_ gas in distilled water (829 µM at 25 °C) at atmospheric pressure, and distilled water was utilized as a negative control. All manipulations were conducted at 25 °C ± 1 °C.

### Determination of thiobarbituric acidreactive substances (TBARS) contents

Lipid peroxidation was estimated by calculating the contents of TBARS as previously described with a slight change^41^. About 0.5 g of fresh tissues were ground in 5% 2-thiobarbituric acid (TBA) using a mortar and pestle and centrifuged at 5000 g for 15 min. Taking 1 ml supernatant and 1 ml 0.67% TBA, mixed, rapid cooling after bathing at 100 °C for 30 min and centrifuged at 3000 g for 10 min. The supernatant was used for calculation of TBARS at 450 nm, 532 nm, 600 nm.

### Antioxidant enzyme activity assays

The antioxidant enzymes were assessed according to the method described by Ara *et al*.^42^ with some modifications. About 2.0 g fresh plant tissues were homogenized in 5 ml of 50 mM potassium phosphate buffer (pH 7.0) for the SOD, POD, APX and CAT assays. The homogenates were centrifuged at 1 2,000 × g for 20 min at 4 °C, and the supernatant was utilized for assays of the enzyme activity. Activities of guaiacol peroxidase (POD) and superoxide dismutase (SOD) were measured at 560 nm and 470 nm, respectively. Catalase (CAT) activity was calculated by observing the consumption of hydrogen peroxide (H_2_O_2_) at 240 nm for a minimum of 3 min. APX activity was estimated by observing the decrease in absorbance at 290 nm within 1 min.

### Real-time quantitative RT-PCR analysis

qRT-PCR was used to examine the gene expression according to the method from Hao *et al*.^43^ with some modifications. Total RNA was isolated from 2.0 g soybean sprouts which were ground in liquid nitrogen until very fine powder was achieved and utilized Trizol reagent to dissolve fine powder (Invitrogen, Gaithersburg, MD, USA) according to the instructions supplied by the manufacturer. Total RNA (8 µL) from varying treatments was reverse-transcribed into cDNA using a cDNA reverse transcription kit (Thermo Fisher Scientific Inc., MA, USA). Transcript levels of several genes weremeasured by qRT-PCR using a Mastercycler® ep realplex real-time PCR system (Eppendorf, Hamburg, Germany) with Bestar® SybrGreen qPCR mastermix (DBI, Bioscience Inc., Germany) according to the manufacturer’s instructions. Primer sequences of cDNA amplification are listed in Supplemental Table 2.

### The content of ascorbic acid and total AsA and the DPPH radical scavenging activity assay

AsA (ascorbic acid) was extracted in metaphosphoric acid without a reducing agent according to a previous method described by Rassam *et al*.^44^ with some modifications. First, 1.0 g of fresh sample was used to make 25 ml of AsA solution in 0.2% metaphosphoric acid. For HPLC analysis, 10 μl of solution was injected into the HPLC Shimadzu LC—20 A auto sampler. A reverse C18 column (InertSustain C18, Shimadzu, Japan) was used and eluted at 1 ml·min^−1^ at 25 °C. The mobile phase was made up of 0.2% metaphosphoric acid in water. Reduced AsA was quantified at its UV absorption maximum of 243 nm. A set of standards containing 2–500 mg·L^−1^ of reduced AsA was made and processed in the same way. AsA concentrations were quantified with standard curve and expressed in µg per g fresh weight. For an assay of AsA+DHA (total AsA), the method was introduced by Huang *et al*.^45^. A portion of the prepared AsA solution above was reduced with freshly made dithiothreitol to a final concentration of 20 mM. The reduction reaction was conducted at room temperature for 6 h, and the reduced samples were measured using the HPLC method described above.

The DPPH radical scavenging activity assay was done according to the method reported by Hsu *et al*.^46^ with some modifications. Briefly, standard solutions was prepared and added to a 70 µmol/L DPPH methanolic solution. Incubation period of 30 min was allowed at room temperature in dark place to complete any reaction that is to be occurred. Then absorbance was measured by UV spectrophotometer at 517 nm. The radical scavenging capacity was expressed as percentage effect (%) and calculated using the Equation 1.

1 $${\rm{Percentage}}\,{\rm{effect}}\,( \% )=(\frac{{{\rm{Abs}}}_{{\rm{control}}}-{{\rm{Abs}}}_{{\rm{sample}}}}{{{\rm{Abs}}}_{{\rm{control}}}})\times 100$$

### Detection of H_2_O_2_ concentration

The H_2_O_2_ concentration was measured colorimetrically as described by Wu *et al*.^47^. H_2_O_2_ was extracted by homogenizing tissue with phosphate buffer (50 mM, pH 6.5) containing 1 mM hydroxylamine. The homogenate was centrifuged at 6,000 g for 25 min. To determine H_2_O_2_ concentration, the extracted solution was mixed with 0.1% titanium chloride in 20% (v/v) H_2_SO_4_. Te mixture was then centrifuged at 6,000 g for 25 min. The absorbance was measured at 410 nm. The NO concentration was calculated by comparing to against a standard curve of H_2_O_2_.

### Statistical analyses

All values are averages of three replicated experiments. The data was subjected to one-way analysis of variance (ANOVA) and varying letters indicate significant differences between the treatments which were compared using the Duncan’s multiple range test (*p* < 0.05). All statistical analysis was conducted using SPSS 19.0 for Windows.

## Electronic supplementary material

Supplementary Information
