# Supplementary material for: Hydrogen gas mediates ascorbic acid accumulation and antioxidant system enhancement in soybean sprouts under UV-A irradiation
Source: Sci Rep. 2017 Nov 27;7:16366. doi: 10.1038/s41598-017-16021-0 (PMC5703957; doi:10.1038/s41598-017-16021-0)
Supplement: Supplementary file 1 — Supplementary Information [file 41598_2017_16021_MOESM1_ESM.docx]

**Type of contribution:** regular papers

**Title:** Hydrogen gas mediates ascorbic acid accumulation and antioxidant system enhancement in soybean sprouts under UV-A irradiation

**Author names:** Li Jia^1^, Jiyuan Tian^1^, Shengjun Wei^1^, Xiaoyan Zhang^1^, Xuan Xu^1^, Zhenguo Shen^1^, Wenbiao Shen^1^, Jin Cui^1^*

**Affiliation:**

^1^College of Life Sciences, Nanjing Agricultural University, Nanjing, Jiangsu 210095,China

***Corresponding author:** Jin Cui; E-mail: cuijin@njau.edu.cn

***Correspondence:** E-mail: cuijin@njau.edu.cn; Telephone number: +86 025 84395920

**Supplementary Materials:**

**
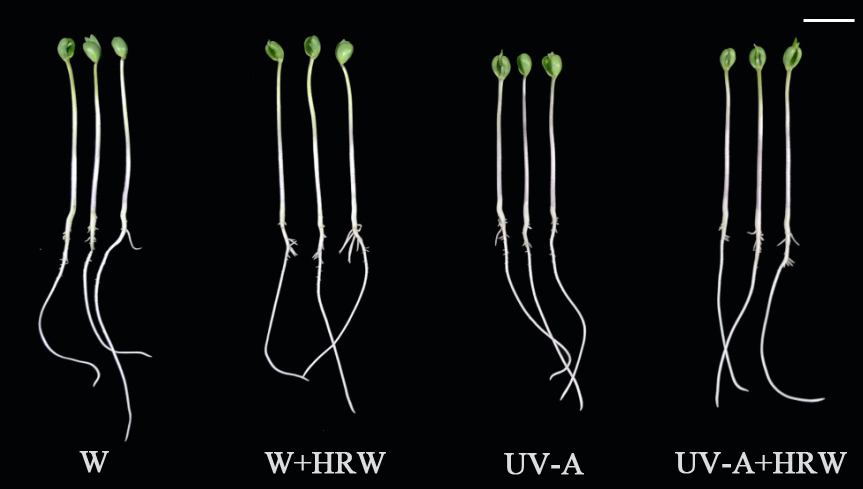
**

**Supplemental Fig 1.** Effects of the different light qualities on the appearance of soybean sprouts. Sprouts cultivated in distilled water (H_2_O) or HRW were exposed to white light or UV-A for 36 h. W: Sprouts cultivated in distilled water under white light; W+HRW: Sprouts cultivated in HRW under white light; UV-A: Sprouts cultivated in distilled water under UV-A; UV-A+HRW: Sprouts cultivated in HRW under UV-A.Bar = 2 cm.

**Supplemental Table 1**

Effects of different light qualities on the appearance of soybean sprouts.

| Treatment | Root length/cm | Hypocotyl length/cm | Total Fresh weight/g | Edible Fresh weight/g | Edible rate/% |
| --- | --- | --- | --- | --- | --- |
| W | 10.51±0.80a | 9.31±0.83a | 0.57±0.05a | 0.45±0.04a | 0.79±0.02a |
| W+HRW | 10.09±1.19a | 8.71±0.82a | 0.56±0.10a | 0.46±0.07a | 0.82±0.03a |
| UV-A | 10.05±0.59a | 6.23±0.33b | 0.50±0.02b | 0.4±0.02b | 0.81±0.01a |
| UV-A+HRW | 10.15±0.52a | 8.13±0.41 a | 0.55±0.05a | 0.44±0.02a | 0.81±0.03a |

**Supplemental Table 2**

The nucleotide sequence of primers used in RT-PCR

| Gene name | | Gene NCBI ID | Prime forward | Prime reverse |
| --- | --- | --- | --- | --- |
| ***Actin*** | **J01298.1** | TCCCAGTATTGTTGGCCGA | TTCCATGTCATCCCAGTTGCT |  |
| ***GMP*** | **FJ792604.1** | AAAACACACGGAGGAGAGGC | CCCGGTAGTCTCCTCCATCA |  |
| ***GME*** | **LOC100806498** | GGCTCCTGCTGCTTTTTGTC | TCTGAGCACCCCTTCAACAC |  |
| ***VTC2*** | **HQ636607.1** | TTGAGAAGGCCCCCACTAAGA | GCCACCCTCAAAGACGAGA |  |
| ***VTC4*** | **LOC100817591** | GTGGCTCCTGTGCTCTGAAT | ACAGCACCACCTGCTACATC |  |
| ***GDH*** | **LOC100809400** | CGCTGGATGTTGTGCTTTCA | TCGGTGAGAAGGCCCATAGA |  |
| ***GLDH*** | **LOC100526948** | ACTCCAGCGGCAATTCTCAT | CGGGAGGGGGAATGTGTAGT |  |
| ***APX1*** | **NM_001250856.1** | TTGACAAGGGCACGAAGACC | TTGAGTGGCTCCAAAAGCCT |  |
| ***APX2*** | **AB082932.1** | CTTACGACGTGAGCTCGAAGA | GCTCCAAAAGCCTAACAGCG |  |
| ***AO*** | **XM_003555611.2** | TAGAGGAAGAAAGCCTAGCACC | ATTCCAAAGGGGTGTGATGGG |  |
| ***MDHAR*** | **AB442087.1** | TGTGGACATGGTTGTGGTGG | TTCGTGTTTCCCCAACTGCT |  |
| ***DHAR*** | **NM_001250000.1** | ACAAGTGCACAACCACAGGA | TGTTGGGGTATAGTCCTCAGAGT |  |
| ***GR*** | **L11632.1** | CGGTGCCTCAAGGGAAGAAAT | TTCACTCGACTTCCAGGCTC |  |
